# Supplementary material for: Aragonite dissolution protects calcite at the seafloor
Source: Nat Commun. 2022 Mar 1;13:1104. doi: 10.1038/s41467-022-28711-z (PMC8888755; doi:10.1038/s41467-022-28711-z)
Supplement: Supplementary file 1 — Supplementary information [file 41467_2022_28711_MOESM1_ESM.pdf]

Supplementary information to "Aragonite dissolution protects calcite at the seafloor"

Olivier Sulpis[1], Priyanka Agrawal[1], Mariette Wolthers[1], Guy Munhoven[2], Matthew Walker[3,4], Jack J. Middelburg[1]
[1]Department of Earth Sciences, Utrecht University, The Netherlands
[2]Département d'Astrophysique, Géophysique et Océanographie, Université de Liège, Belgium
[3]School of Life Sciences, University of Lincoln, United Kingdom
[4]Leeds Institute of Data Analytics (LIDA), University of Leeds, United Kingdom
Corresponding author: Olivier Sulpis (o.j.t.sulpis@uu.nl)

Supplementary Table 1. Initial composition of water. Initial concentrations and total activity coefficients are from PHREEQC. Diffusion coefficients are from [a] (Yuan-Hui Li & Sandra Gregory, 1974) and [b] (Horst D. Schulz, 2006) at 25°C.

**Variable**	**Initial concentration** (c_i, mol m^{-3})	**Diffusion coefficient** (D_i, m^2 s^{-1})	**Total activity coefficient** (γ_i, unitless)
$[H^+]$	1.166×10^{-4}	[a]9.31×10^{-9}	0.755
$[OH^-]$	1.913×10^{-4}	[a]5.27×10^{-9}	0.611
$[H_2CO_3^*]$	1.470×10^{-1}	[b]1.79×10^{-9}	1.164
$[HCO_3^-]$	1.493	[a]1.18×10^{-9}	0.676
$[CO_3^{2-}]$	3.717×10^{-3}	[a]0.96×10^{-9}	0.209
$[Ca^+]$	10.85	[a]0.79×10^{-9}	0.251
$[Na^+]$	497.4	[a]1.33×10^{-9}	0.706
$[Cl^-]$	579.2	[a]2.03×10^{-9}	0.625
$\Omega_{calcite}$	0.64		
$\Omega_{aragonite}$	0.46		
pH	7.05		

Supplementary Table 2. Grain properties: species name, material, surface area, weight, specific surface area and major axis length, i.e., the widest diameter of the shell.

**Name**	**Material**	**Surface area** (mm^2)	**Volume** (mm^3)	**Weight** (µg)	**SSA** ($m^2\ g^{-1}$)	**Major axis** (µm)
Conceptual spheric grains						
Calcite sphere	Calcite	0.28	0.0141	37.94	0.0074	~300
Aragonite sphere	Aragonite	2.01	0.2681	785.24	0.0026	~800
Foraminifera shell scans						
G. nepenthes	Calcite	1.32	0.0226	61.25	0.0216	~620
M. menardii	Calcite	3.28	0.0839	227.37	0.0144	~1180
B. adamsi	Calcite	0.20	0.0034	9.21	0.0217	~380
Pteropod shell scans						
H. inflatus	aragonite	2.49	0.0227	66.51	0.0374	~850

Supplementary Table 3. Summary of the different model simulations and their settings.

**Simulation name**	**Grains involved**	**Sinking speed *u*** ($m\ d^{-1}$)	**Dimensions** (mm×mm×mm) x × y × z	**Moving mesh**	**Boundary conditions**
Static A	*G. nepenthes*	0	4×4×4	No	Periodic
Static B	*G. menardii*	0	4×4×4	No	Periodic
Static C	*G. adamsi*	0	3×3×3	No	Periodic
Static D	*H. inflatus*	0	3×3×3	No	Periodic
Sediment A	Calcite spheres	0	3.15×3.15×3.5	No	Periodic, no flux, fixed
Sediment B	Calcite spheres + *H. inflatus*	0	3.15×3.15×3.5	No	Periodic, no flux, fixed
Shrinking A	Aragonite sphere	0	3×3×3	Yes	Periodic
Shrinking B	Aragonite sphere	100	3×3×6	Yes	Periodic, no flux
Shrinking C	Aragonite sphere + calcite spheres	0	3.15×3.15×2.55	Yes (for aragonite)	Periodic, no flux, fixed

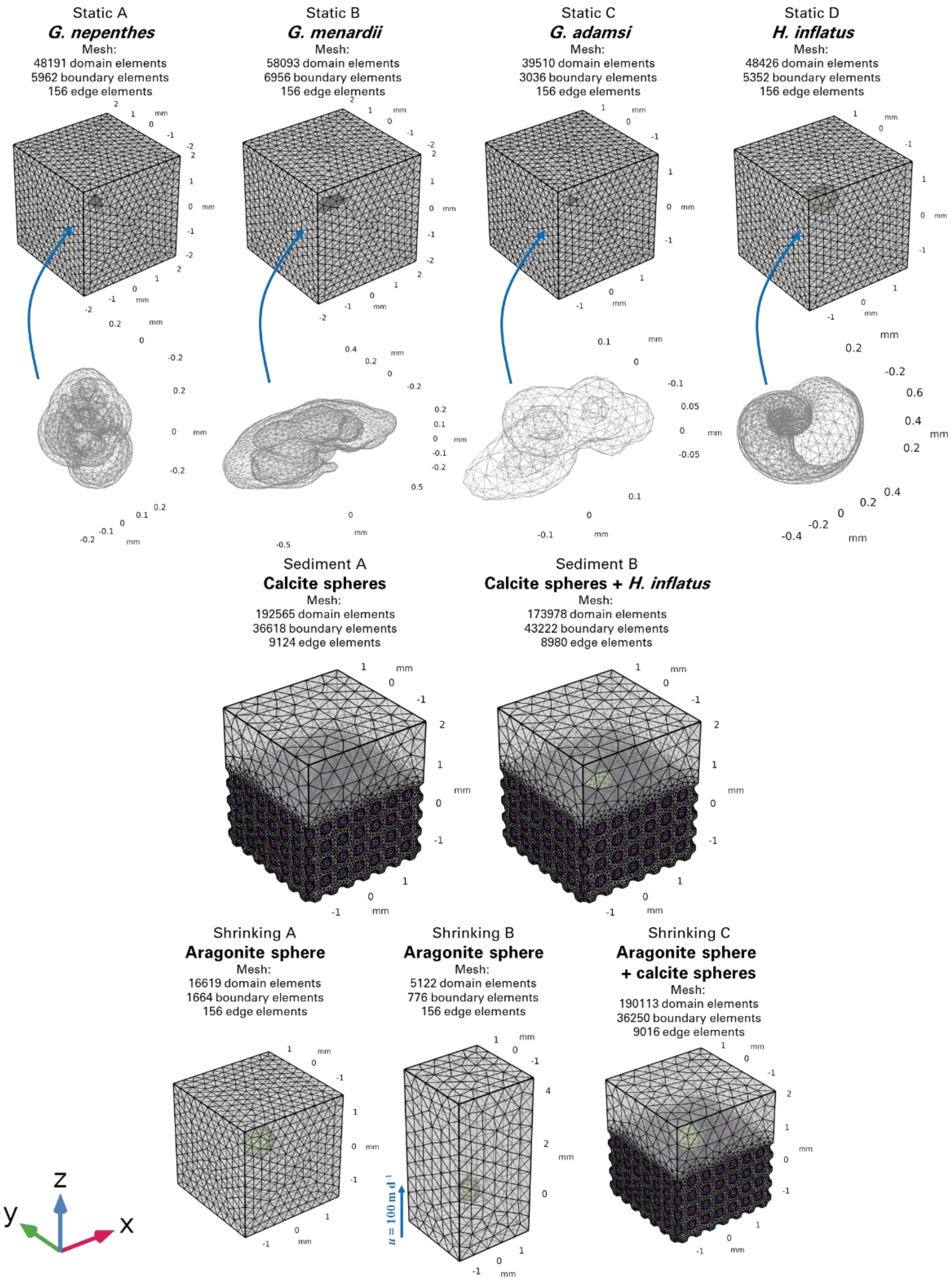

Supplementary Figure 1. Summary of the different model simulations and visualization of their meshs. The top row shows the four "static" simulations in which natural $CaCO_3$ grains dissolve suspended in a volume of water. The middle row shows the two "sediment" simulations aiming to identify the role of aragonite dissolution at the seafloor on porewater chemistry and the dissolution of surrounding calcite grains. The bottom row represents the three "shrinking" simulations in which the aragonite grain mesh was reevaluated at each time step to account for the weight loss due to dissolution. Magenta color represents calcite surfaces while bright green stands for aragonite surfaces.

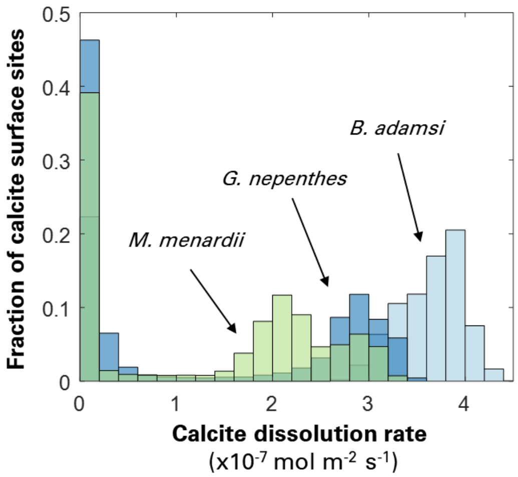

Supplementary Figure 2. Bimodal distribution of foraminiferal calcite dissolution rates across the dissolving shell surface. The three foraminifera species B. adamsi (in yellow), G. nepenthes (in orange) and M. menardii (in blue) are shown in Fig. 1.

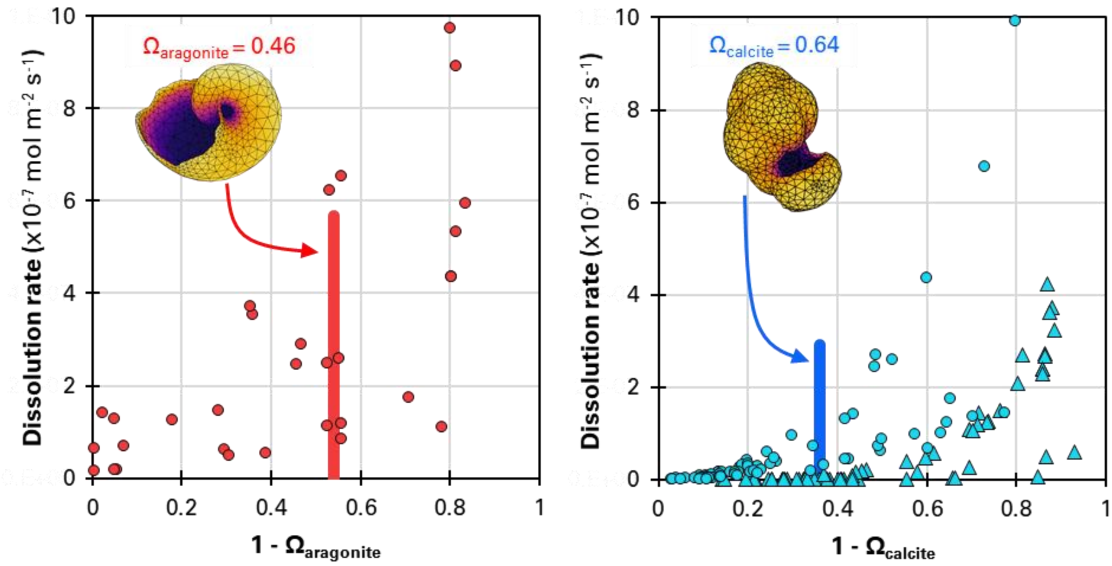

Supplementary Figure 3. Steady-state grain surface area-normalized calcite (in blue) and aragonite (in red) dissolution rates as a function of the steady-state undersaturation state with respect to aragonite ($1 - \Omega_{aragonite}$) or calcite ($1 - \Omega_{calcite}$). Dots represent experimental "bulk" dissolution rates from the literature (Lynn M. Walter & John W. Morse, 1985; Robin S. Keir, 1980), in which the measured dissolution rates are normalized by the total area (in m^2) of the grains. Lines represent the dissolution rates as parameterized in the present study, in which the [m^{-2}] in the dissolution rate expression refers to an elementary surface area unit. Aragonite and calcite dissolution rates were recomputed using original data from (Lynn M. Walter & John W. Morse, 1985; Robin S. Keir, 1980). From these two publications, all the dissolution rates from biogenic and synthetic samples were used. Units for the dissolution rates were changed to [mol m^{-2} s^{-1}] using the specific surface areas for each grain as reported in the original publications. Dissolution rates for synthetic aragonite were not reported because most of them were obtained in seawater supersaturated with respect to aragonite, thus we suspect the synthetized material was not aragonite. Saturation states were recomputed using the ionic concentration products at steady state measured by the authors, and dividing by the stoichiometric solubility constant with respect to either aragonite or calcite from (Alfonso Mucci, 1983) at the salinity and temperature of experiments, to harmonize the data.

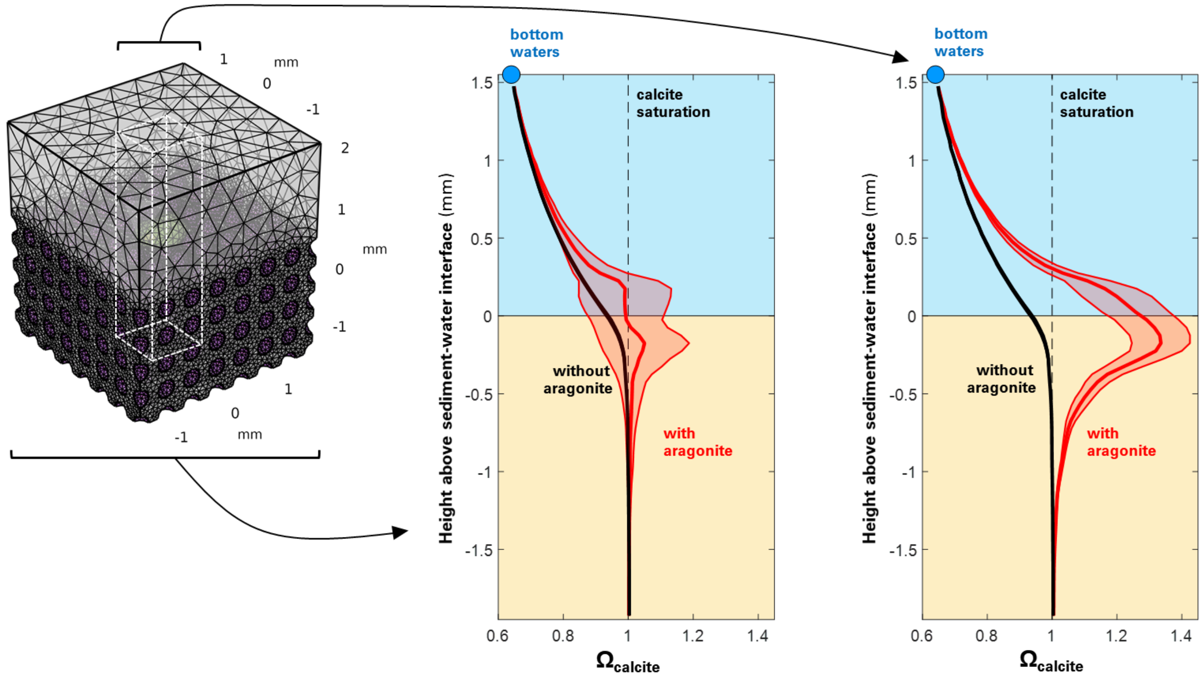

Supplementary Figure 4. Depth profiles of saturation state with respect to calcite. The blue circle represents the bottom-water value, above the diffusive boundary layer. The black depth profile stands for a case without a pteropod, in which it is replaced by four calcite spheres evenly spaces one from another, while the red depth profiles represent the situation with aragonite shown on the right and in Fig. 4. The depth profile in the middle refers to the whole sediment volume whereas the depth profile on the right refers to the column just above the pteropod.

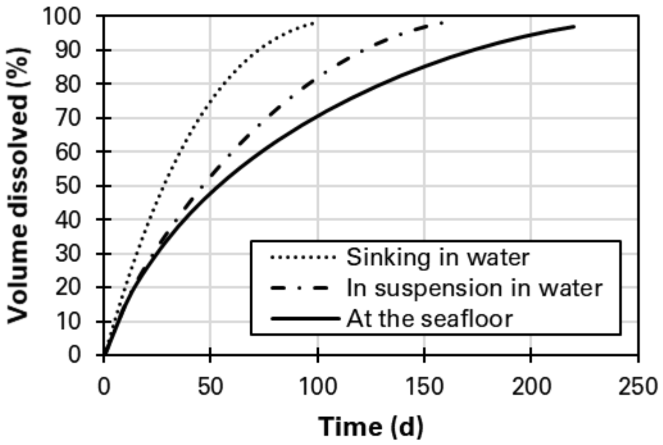

Supplementary Figure 5. Dissolution of aragonite spheres as a function of time. The simulations did not run until full dissolution because of the increasing difficulty to produce a mesh for dissolving particles as they become smaller. The dotted line represents dissolution for an aragonite sphere sinking through water, the dash/dot line stands for dissolution of a particle in suspension in water, and the solid line is the case for an aragonite sphere sitting above a calcite-spheres sediment.

Supplementary references

Alfonso Mucci. (1983). The solubility of calcite and aragonite in seawater at various salinities, temperatures and one atmosphere total pressure. *American Journal of Science*, *283*(7), 780–799.

Horst D. Schulz. (2006). Quantification of Early Diagenesis: Dissolved Constituents in Pore Water and Signals in the Solid Phase. In H. D. Schulz & M. Zabel (Eds.), *Marine Geochemistry*. Berlin, Heidelberg: Springer.

Lynn M. Walter, & John W. Morse. (1985). The dissolution kinetics of shallow marine carbonates in seawater: A laboratory study. *Geochimica et Cosmochimica Acta*, *49*(7), 1503–1513.

Robin S. Keir. (1980). The dissolution kinetics of biogenic calcium carbonates in seawater. *Geochimica et Cosmochimica Acta*, *44*(2), 241–252.

Yuan-Hui Li, & Sandra Gregory. (1974). Diffusion of ions in sea water and in deep-sea sediments. *Geochimica et Cosmochimica Acta*, *38*(5), 703–714.
